# Cold Atmospheric Plasma Ameliorates Skin Diseases Involving Reactive Oxygen/Nitrogen Species-Mediated Functions

**DOI:** 10.3389/fimmu.2022.868386

**Published:** 2022-05-26

**Authors:** Si-yue Zhai, Michael G. Kong, Yu-min Xia

**Affiliations:** ^1^ Department of Dermatology, The Second Affiliated Hospital of Xi’an Jiaotong University, Xi’an, China; ^2^ Center of Plasma Biomedicine, State Key Laboratory of Electrical Insulation and Power Equipment, Xi’an Jiaotong University, Xi’an, China; ^3^ School of Electrical Engineering, Xi’an Jiaotong University, Xi’an, China

**Keywords:** cold atmospheric plasma, reactive oxygen species, reactive nitrogen species, skin disease, therapy

## Abstract

Skin diseases are mainly divided into infectious diseases, non-infectious inflammatory diseases, cancers, and wounds. The pathogenesis might include microbial infections, autoimmune responses, aberrant cellular proliferation or differentiation, and the overproduction of inflammatory factors. The traditional therapies for skin diseases, such as oral or topical drugs, have still been unsatisfactory, partly due to systematic side effects and reappearance. Cold atmospheric plasma (CAP), as an innovative and non-invasive therapeutic approach, has demonstrated its safe and effective functions in dermatology. With its generation of reactive oxygen species and reactive nitrogen species, CAP exhibits significant efficacies in inhibiting bacterial, viral, and fungal infections, facilitating wound healing, restraining the proliferation of cancers, and ameliorating psoriatic or vitiligous lesions. This review summarizes recent advances in CAP therapies for various skin diseases and implicates future strategies for increasing effectiveness or broadening clinical indications.

## Introduction

It is widely accepted that there are various kinds of skin diseases, including infectious diseases, cancers, wounds, and non-communicable inflammatory skin diseases (ncISDs). The pathogenic factors of these skin disorders include microbial infections, excessive cytokines release, inflammatory cell infiltration, and aberrant cell proliferation or differentiation (1–3). Currently, the comprehensive treatments include systemic or topical medications or in combination with different phototherapies. However, these applications have still been challenged due to systemic side effects, the high recurrence rate of the disease, or the serious impact on daily life or working (4). As an innovative non-invasive application, cold atmospheric plasma (CAP) has exhibited significant efficacy in treating skin diseases such as microbial infections, cutaneous wounds, cancers, and ncISDs (5, 6). It is well clarified that the biological functions of CAP involve ultra-violet radiation, reactive oxygen species, and reactive nitrogen species while exhibiting little damage to the normal cells or tissues (7). CAP may destroy biofilms or even the integrity of cells, degrade cellular proteins and DNA, generate DNA and protein crosslinking, and dysregulate pH value- or calcium-related microenvironments (7–11). In this review, we summarize recent advances in the applications of CAP in treating skin diseases and possible therapeutic mechanisms.

## The Characteristics of CAP

**Figure 1:**
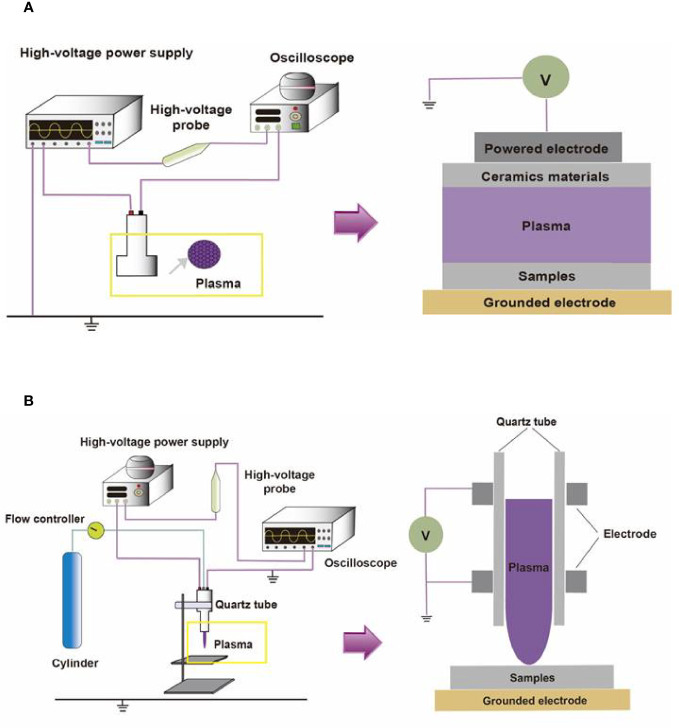
The compositions of two different CAP platforms. **(A)** The surface barrier discharges or** (B) **the atmospheric pressure plasma jet consists of a high-voltage source, high-voltage probe, oscilloscope, quartz tube, cylinder, and flow controller.

**Figure 2:**
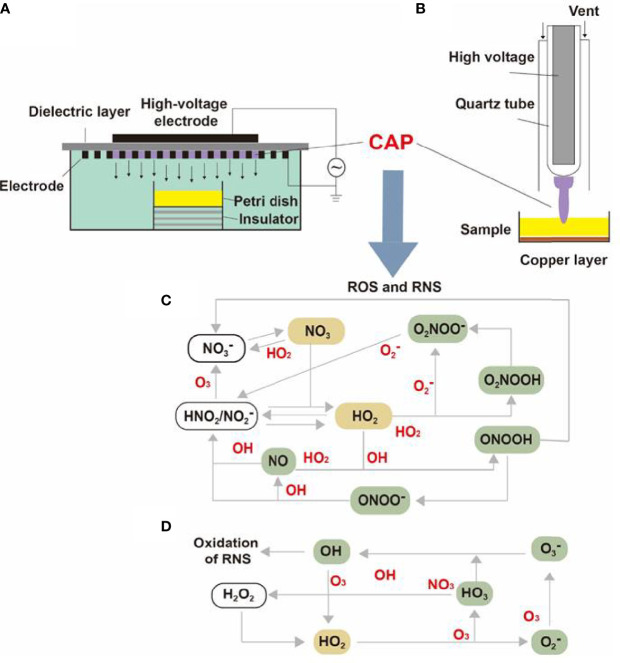
The internal structure of surface DBD and APPJ and the transformation of CAP particles. The internal structures of **(A)** surface DBD and **(B)** APPJ were consisted of electrodes, high-voltage probes, quartz tubes, and a dielectric layer. The **(C)** RNS and **(D)** RNS generated under high voltage could be converted into different molecules or ions and finally synthesized into effective particles like ^1^O_2_, ONOO^-^, NO_2_^-^, NO_3_^-^, and H_2_O_2_.

**Figure 3:**
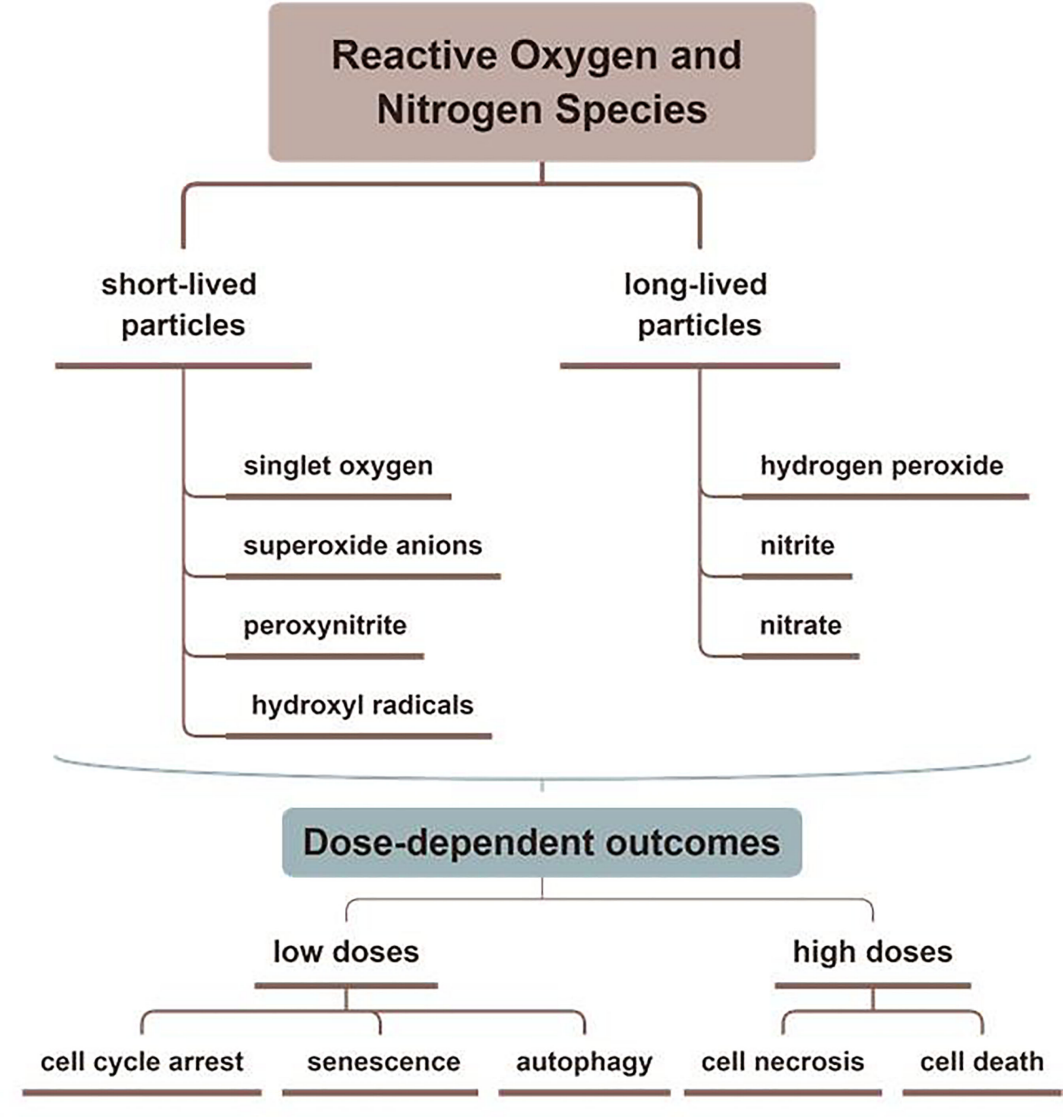
The typical functions of ROS and RNS in CAP. Various kinds of effective particles, including ROS and RNS, are divided into short-lived or long-lived substances, which display different mechanisms in treating skin diseases by CAP.

Containing with the positive and negative changed species, plasma is the fourth state of matter, which is different from the common three substances (solid, liquid, and gas) (12). According to the gas temperature of plasma, the plasma generated by different methods can be divided into hot plasm, warm plasma, and cold plasma. Because high temperature can cause thermal damage to organisms, the plasma applied to medical treatment should be considered a kind of plasma at atmospheric pressure with its temperature close to room temperature (13). CAP has been used in medical treatments widely (6). As **Figure 1** described, there are two common devices for CAP generators, dielectric barrier discharges (DBD) and atmospheric pressure plasma jet (APPJ). Surface DBD is consisted of two parallel electrodes sandwiching a ceramic slab to strike a surface discharge plasma at a high voltage in a narrow discharge gap (7). The other is the APPJ, consisting of a quartz capillary equipped with a pin-type electrode at a high voltage generated in an open space (12). In order to improve the efficiency of CAP, helium (He), argon (Ar), nitrogen (N_2_), oxygen (O_2_), artificial air, and two or more mixtures of these gases can be utilized to generate CAP (14). It is documented that reactive oxygen species (ROS) (15) or reactive nitrogen species (RNS) (16), play an essential role in CAP application in dermatology. **Figure 2** has shown the interconverting ROS and RNS that could be classified into short-lived and long-lived species, such as singlet oxygen (^1^O_2_), superoxide anions (O_2_^-^), hydroxyl radicals (^•^OH), hydrogen peroxide (H_2_O_2_), nitric oxide (NO), peroxynitrite (ONOO^-^), nitrite (HNO_2_/NO_2_^-^) and nitrate (HNO_3_/NO_3_^-^). ^1^O_2_ is a highly reactive molecule that participates in apoptosis processes and produces some oxidants and cytotoxic molecules. O_2_^-^ has relatively high reactivity and results in oxidative stress in cells to generate hydroxyl radicals. It is displayed that ^•^OH belongs to extremely reactive oxidizing species, which can damage the lipid peroxidation, protein, membrane and result in cell death. As for ONOO^-^, it reacted with H_2_O_2_ to generate ^1^O_2_, which inhibited the catalase activity of tumor cells, resulting in the accumulation of H_2_O_2_ in the part of the peripheral environment. Then H_2_O_2_ entered tumor cells through aquaporin on the cell surface and consumed internal glutathione, which could promote the apoptosis of tumor cells *via* lipid peroxidation (17, 18). Besides, as a long-lived species and second messenger for signaling cascades, H_2_O_2_ can inhibit the activity of microbes and kill them directly (19). It has been documented that CAP-induced changes in target cells are often closely related to intervention time, voltage, and other factors. For example, CAP could induce cell cycle arrest, senescence, and autophagy at low doses, but high doses of CAP could lead to irreversible cell necrosis (20). Moreover, RNS has been confirmed to regulate cell differentiation, self-renewal, and the balance between quiescence and proliferation (21). The occurrence and development of tumors can be influenced by NO concentration, which can accelerate tumor growth in the low-level concentration but inhibit it at high levels (22). The typical functions of ROS and RNS have been presented in **Figure 3**.

## ROS and RNS Inactivated Microorganisms by Destroying Cell Biofilms and Components

**Figure 4:**
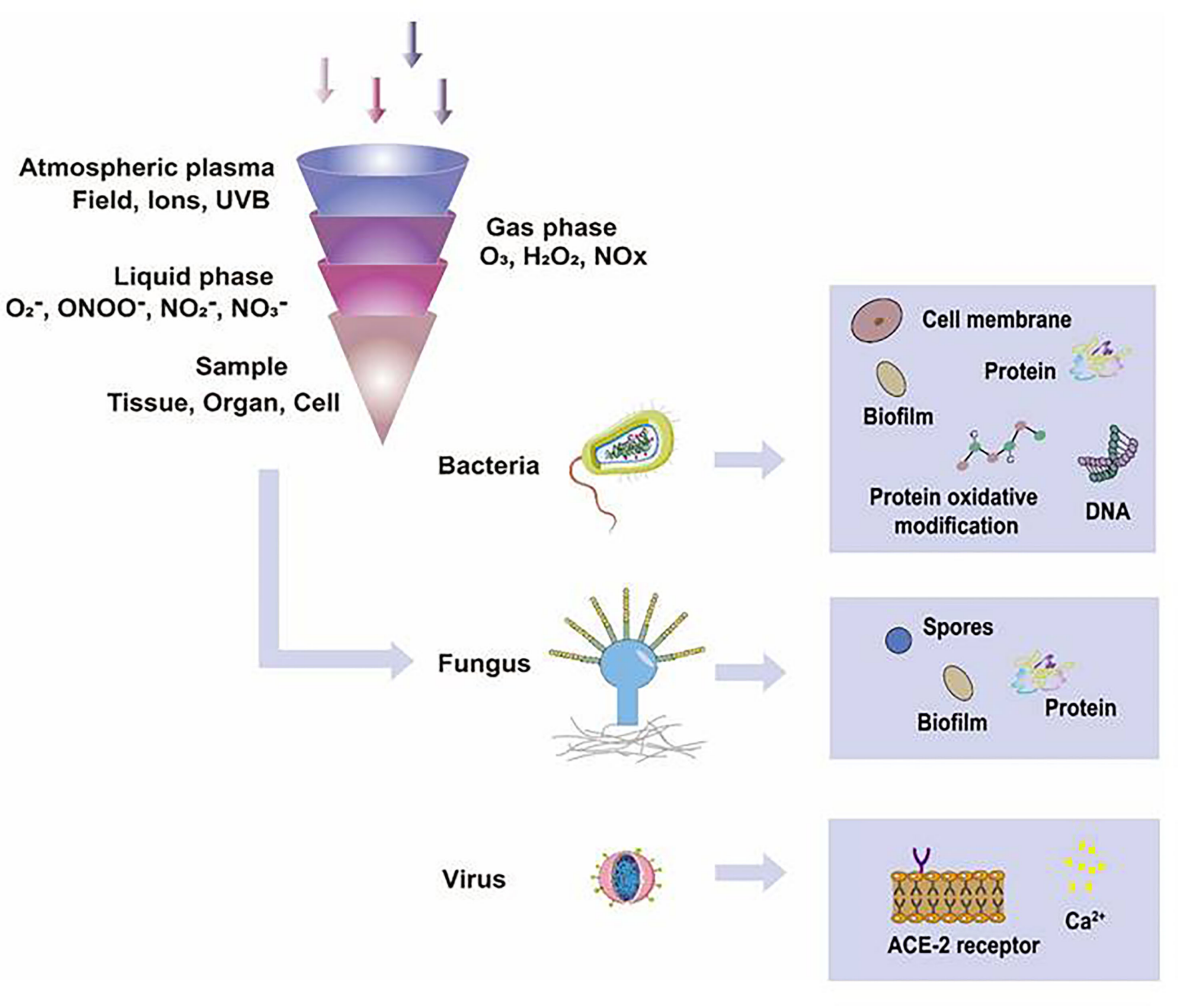
The functions of CAP on microorganisms. In different gas or liquid media, CAP could produce different ROS and RNS, such as ^1^O_2_, ONOO^-^, NO_2_^-^, NO_3_^-^, and H_2_O_2_, which inactivated microorganisms effectively. The possible mechanisms that CAP applied in inactivating organic samples included destroying biofilms, damaging cell membranes, degrading DNA, RNA, and proteins, changing the chemical modification of proteins, and directly leading to the death of microorganisms.

It is well displayed that CAP has shown promising effects in inactivating microorganisms (**Figure 4**). CAP can inhibit the proliferation of microorganisms on the surface of medical devices but prohibit the infection of various microorganisms, including *Staphylococcus aureus*, *Streptococcus pyogenes*, *Pseudomonas aeruginosa*, *Yersinia enterocolitica*, MRSA, and MRGN (8, 23, 24). In addition, related studies have reported that CAP has satisfactory efficacy in treating the pathogens of infectious skin diseases, such as *Trichophyton rubrum*, *Mycobacterium canis*, and *Candida albicans* (**Table 1**) (8, 25). In this section, we will explore the inactivation mechanisms of CAP against bacteria, fungi, and viruses, respectively.

**Table 1:** The mechanisms of CAP for inactivating pathogens.

Bacteria	Time	Device	Gas	Target
*E. faecalis*	2019	DBD	O_2_	Biofilm
*L. monochromous*	2019	DBD	He	Biofilm
*S. typhimurium*	2019	DBD	He	Biofilm
*S. bacteria*	2014	APPJ	Air	Biofilm
*P. aeruginosa*	2014	APPJ	Air	Biofilm
*K. pneumonia*	2014	APPJ	Air	Biofilm
*S. aureus*	2014 2012	APPJ DBD	He Air	Biofilm Membrane
*P. fluorescens*	2014 2012	APPJ DBD	He Air	Biofilm Membrane
*S. epidermidis*	2011	DBD	Air	Biofilm DNA
*E. coli*	2018 2017	DBD APPJ	He + air He + O_2_	Protein DNA DPC
**Fungus**	**Time**	**Device**	**Gas**	**Target**
*C. albicans*	2012	APPJ	Argon Argon + O_2_	Biofilm
*T. rubrum*	2013	DBD	Air	Biofilm
*M. canis*	2015 2013	APPJ DBD	Air Air	Biofilm Protein Spores
*E. floccosum*	2015	APPJ	Air	Protein Spores
*M. gypseum*	2015	APPJ	Air	Protein Spores
**Visus**	**Time**	**Device**	**Gas**	**Target**
hAV	2018	DBD	Argon	Protein DNA
COVID-19	2021 2022	APPJ APPJ	He Argon	Ca^2+^ ACE2

E. faecalis, Enterococcus faecalis; L. monochromous, Listeria monochromous; S. typhimurium, Salmonella typhimurium; S. bacteria, Salmonella bacteria; P. aeruginosa, Pseudomonas aeruginosa; K. pneumonia, Klebsiella pneumonia; S. aureus, Staphylococcus aureus; P. fluorescens, Pseudomonas fluorescens; S.epidermidis, Staphylococcus epidermidis; E. coli, Escherichia coli; DPC, DNA and protein crosslinking; C. albicans, Candida albicans; T. rubrum, Trichophyton rubrum; M. canis, Microsporum canis; E. floccosum, Epidermophyton floccosum; M. gypseum, Microsporum gypseum; hAV, Human adenovirus; COVID-19, coronavirus disease-19; ACE2, angiotensin-converting enzyme 2; DBD, dielectric barrier discharge; APPJ, atmospheric pressure plasma jet.

### Bacteria

Generally speaking, bacterial biofilms have widely existed on the surfaces of various foods, medical devices, and human tissues or organs under pathological conditions. Due to the widespread use of antibiotics, opportunistic pathogen infections caused by bacterial biofilms have gradually increased. Once a biofilm is formed, the organism will resist the action of antibiotics, making it difficult for antibiotics to remove bacterial biofilms or kill isolated bacteria on the organism’s surface. Due to its anti-biofilm properties, CAP has been widely used in sterilization (26, 27). First, CAP can accelerate the death of bacteria *via* wrecking bacterial biofilms. Theinkom used SMD to treat Enterococcus faecalis to investigate the potential effects of CAP on microbial biofilms with 3.5 kV, 4.0 kHz, and 0.5-1 W/cm^2^. And the results showed that CAP treatment for 1 minute could inactivate bacterial biofilms and cause bacterial death directly. Moreover, a 10-min CAP intervention showed higher inactivating efficiency than a 5-min intervention (28). Utilizing a DBD with helium gas, Govaert treated the biofilms of *L. monochromous* and *S. typhimurium* for different minutes to explore the sterilization effects and mechanisms of CAP application by detecting the density of remaining cells (29). Scientists have confirmed the inactivating effects of CAP on bacterial biofilms formed by bacteria, such as *S. bacteria*, *P. aeruginosa*, *K. pneumoniae*, *E. faecalis*, *S. aureus*, *L. monochromous*, and *P. fluorescens* (30–33). Guo and her teams utilized a 6-min CAP intervention to treat *S. aureus* to explore the mechanisms of CAP inactivating microbial biofilms. Their results showed that ROS or RNS from CAP could result in biofilm lysis and bacterial death by promoting the oxidative modification of bacterial nucleoproteins, accelerating the filamentous temperature-sensitive Z of *S. aureus* (SaFtsZ) to lose its assembly ability, and disrupting the activity of ATP-dependent caseinolytic protease P subunit of *S. aureus* (SaClpP) (34). In conclusion, CAP can expand the ROS/RNS binding area, enhance the oxidative modification of cellular proteins and promote the fusion of microbial biofilms with extracellular matrix components, which triggers the degradation of microbial biofilms and inhibits the proliferation of microorganisms.

Second, the changes in cell composition and microenvironment also affect bacterial inactivation. After DBD treatment, Helmke confirmed that the extracellular environment of bacteria was significantly acidified, and the cell membrane of bacteria gradually collapsed. After a while, the cell membrane of bacteria was destroyed, and its DNA was rapidly broken down (11). The destruction of bacterial DNA is the cause of bacterial inactivation, but the modification of bacterial proteins is considered a significant cause of bacterial inactivation. After DBD treatment, Helmke confirmed that the extracellular environment of bacteria was significantly acidified, and the cell membrane of bacteria gradually collapsed. After a while, the cell membrane of bacteria was destroyed, and its DNA was rapidly broken down (11). Although the exact type of DNA damage has not been identified, it is well documented that ROS and RNS from CAP can destroy bacterial DNA directly (35, 36). The destruction of bacterial DNA is the cause of bacterial inactivation, but the modification of bacterial proteins is also considered a significant cause of bacterial inactivation. Dezest’s study aimed to explore the interactions between active substances from CAP and the activity and proliferation of E. coli. Their results demonstrated that CAP could produce bacterial inactivation through selective carbonylation of proteins (37). Of course, CAP can also produce the interaction of DNA and proteins to inactivate bacteria. Guo, with her faculties, indicated that the CAP could generate DNA and protein crosslinking (DPC) *in vitro*. They used a DBD device to intervene in E. coli with the gas mixture of helium and artificial air. By analysis of mass spectrometry and hydroperoxide, the results indicated that the formation of DPC caused by ^1^O_2_ from CAP was the principal mechanism of DNA damage (7). In addition, other studies also documented that the shape of bacteria and the thickness of the bacterial cell wall might also be relevant to the CAP inactivation of bacteria (38–40). Taken together, CAP could inhibit bacterial infection by attacking the bacterial biofilms, destroying the integrity of the cell membrane, and inducing the selective carbonylation protein, DNA degradation, and protein crosslinking. The phenomenon could be explained by the mutual transformation and synergy of the ROS/RNS by CAP. The inactivation of CAP for the main pathogenic bacteria in the skin has been shown in **Table 2**.

**Table 2:** The inactivation of CAP for the main skin pathogenic microorganisms.

Microorganism	Skin disease	Skin lesion	Device	Target
*S. aureus*	Impetigo	VesiclesBullousBlisters	APPJDBD	Biofilm
*C. albicans*	Tinea	ItchyWhite PlaquesErythema	APPJ	Biofilm
*T. rubrum*	Tinea capitis, Tinea corporis, Tinea manus	ErythemaScalyItchy	DBD	Biofilm
*M. canis*	Tinea capitis, Tinea corporis, Tinea manus	ErythemaScalyItchy	APPJDBD	Biofilm Protein Spores
*M. gypseum*	Tinea capitis, Tinea manus	ErythemaScalyItchy	APPJ	Protein Spores

Staphylococcus aureus, S. aureus; Candida albicans, C. albicans; Trichophyton rubrum, T. rubrum; Microsporum canis, M. canis; Microsporum gypseum, M. gypseum; DBD, dielectric barrier discharge; APPJ, atmospheric pressure plasma jet.

### Fungus and Virus

A series of chemical processes based on ROS and RNS have been considered the main reason for the destruction of fungal biofilm (40). Fricke, with his faculties, confirmed that the biofilm of C. albicans could be wrecked by CAP (41). Heinlin et al. used CAP application to assess its efficacy against the dermatophytes such as *T. rubrum* and *M. canis in vitro*. The fungal growth was then measured after CAP treatment. The results proved that CAP exhibited efficient effects on the decontamination of *T. rubrum* and *M. canis* (42). Ouf et al. used CAP to treat five types of skin fungus- *E. floccosum*, *M. canis*, *M. gypseum*, and *T. rubrum*. They found that the fungal cell wall had been damaged under the scanning electron microscope, which indicated that CAP exhibited therapeutic effects in inhibiting fungal growth. Furthermore, they also indicated that the CAP could ameliorate symptoms of fungal skin diseases by the generation of NO, which could inhibit the activity of fungal spores *via* reducing the activity of keratinase and increasing the mycelium permeability (43). Generally speaking, ROS and RNS from CAP could destroy fungal spores and the integrity of cell membrane to inhibit the growth of fungus.

Initially, research on whether CAP could effectively inactivate human pathogenic virus was mainly based on the studies conducted in different phage models (8). Yasuda et al. studied the effects of CAP treatment on λ phage. They utilized the DBD device to treat λ phage for 20 seconds. Then they found that due to the rapid denaturation of its protein, the virus was inactivated totally. Their study noted that CAP inactivated viral proteins through denaturation or chemical/physical modification (44). Aboubakr et al. supported that the degradation of viral capsid protein might be the primary mechanism by which CAP caused viral inactivation. Their study showed that CAP treatment for 2 min could wreck the capsid protein (45). From the studies mentioned above, the degradation of viral protein could be the responsible mechanism for CAP-dependent virus inactivation. Compared with protein degradation, was the mechanism by which CAP inactivated viruses included the destruction of viral DNA or RNA by CAP? Guo utilized CAP to treat water-containing bacteriophages T4, Φ174, and MS2. According to the analysis of the genetic materials of bacteriophages, CAP could destroy both their nucleic acids and proteins *via* ^1^O_2_ (46). Besides the viral protein and DNA/RNA, the unique structure “envelope” should also be considered as the third potential mechanism. CAP is more conducive to suppressing non-enveloped viruses (47). HAV is a kind of non-enveloped dsDNA virus. Zimmermann et al. studied the inactivating effect of CAP on HAV. After CAP treatment for 240 seconds, they found that the virus was inactivated efficiently. Moreover, before infecting CMS-5 cells, HAV, eGFP, and firefly luciferase, were treated by CAP. The results showed that the infectivity and replication of HAV were inhibited directly. They believed that the effect of inactivating HAV was mainly due to the RNS from CAP, which could destroy viral proteins and DNA (48). Virus infection of eukaryotic cells was complicated, including multiple signaling mechanisms inside and outside (49). The COVID-19 virus has spread worldwide in the past two years, and people’s health is facing a considerable threat (50). To explore the interaction between CAP and COVID-19 virus, we selected surface discharge plasma to inactivate the COVID-19 pseudovirus. We utilized CAP with 8.36 kV, 23 kHz, and 0.25 W/cm^2^ to treat deionized water to generate solutions including ROS and RNS. Then a COVID-19 pseudovirus incorporated with SARS-CoV-2S protein was recognized as the virus model. The results showed that the short-lived species from CAP-activated deionized water, such as ONOO^–^ and O_2_^–^, could modify the amino acids in proteins, inactivate S protein and prevent the binding between receptor-binding domain and angiotensin-converting enzyme 2 (ACE2) to achieve the disinfection of COVID-19 virus (25). As we all know, developing a new coronavirus vaccine is crucial in helping humans defend against the inflammatory outbreak caused by kinds of viruses. A recent article demonstrated that CAP could promote ROS accumulation in host cells, leading to the lipid peroxidation of the cell membrane, increasing the influx of Ca^2+^, and activating selective autophagy. The phenomenon caused by CAP would accelerate the entry of host cells into G1 and stop at the G2/M stage to increase the nutrients for viral multiplication, which was conducive to the development of new vaccines and clinical treatments (51). Furthermore, although vaccines that treat SARS-CoV-2 have been produced, the continuous mutation of SARS-CoV-2 may reduce the specificity of the vaccines. Dai, with his faculties, explored the effect of CAP on mutant SARS-CoV-2 viruses. Their results confirmed that the interaction of ^•^OH and other substances produced by CAP or CAP-activated medium (PAM) could trigger the nuclear translocation of the viral receptor ACE2 and inhibit the SARS-CoV-2 virus to invasive host cells (52). Due to the different complexity of virus particles, viruses infect host cells through a series of strategies, resulting in physical interactions between host cells and viruses. The ROS and RNS produced by CAP can promote the inactivation of the viruses by destroying the virus envelope, degrading their DNA or protein, and modifying the protein binding site.

## ROS and RNS Accelerated Wound Healing *Via* Cells’ Proliferation, Growth Factors’ Secretion, and Vessels’ Generation

Recent studies have demonstrated that CAP can promote wound healing by regulating cell viability, proliferation, migration, and inflammatory reaction (53–55). As a secondary messenger, H_2_O_2_ could drive redox signaling pathways and participate in the steady-state, inflammation, proliferation, and remodeling stages of wound healing (56). In addition, ROS and RNS also acted as the mediator of various cellular reactions, affecting cell differentiation, cell apoptosis, cell structure, and the structural integrity of connective tissue (57–60). Shome used a co-culture model to study the effect of CAP on wound healing and paracrine crosstalk between keratinocytes and fibroblasts *in vitro*. They observed that CAP treatment resulted in the up-regulation of the HIPPO transcription factor YAP in HaCaT and fibroblasts. As downstream effectors of the HIPPO signaling pathway, CTGF and Cyr61 were also up-regulated in their studies (56). In conclusion, their results confirmed that at the beginning of wound healing, CAP activated regeneration signaling pathways and stimulated communication between skin fibroblasts and keratinocytes, which promoted the healing of keratinocyte wounds co-culture model *in vitro*.

**Figure 5:**
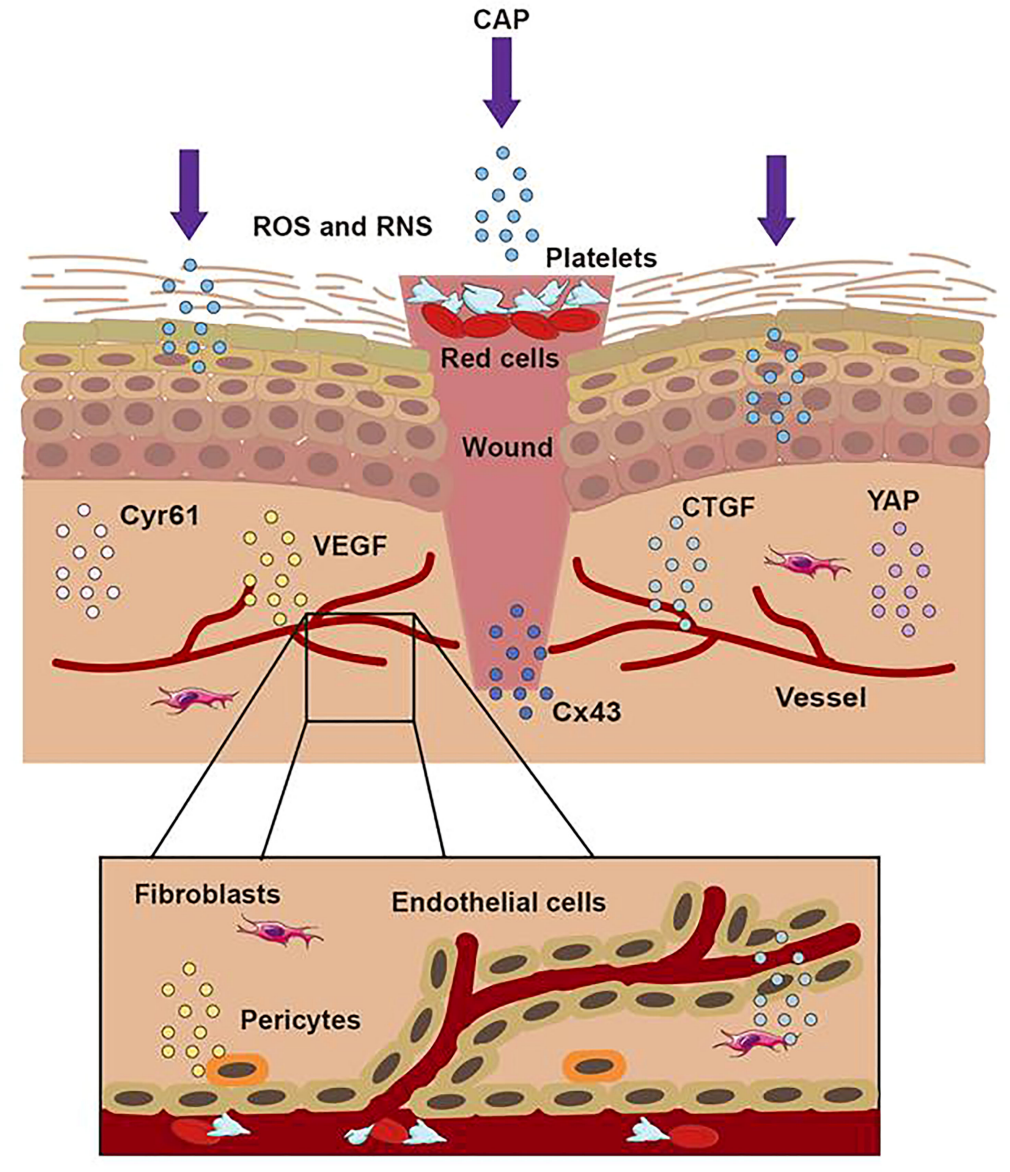
The treatment of CAP on wound. When the skin was hurt, the first step was to form a blood scab to protect the wound. CAP could accomplish wound healing through short-lived and long-lived ROS and RNS. CAP could promote the formation of new blood vessels, strengthen the release of CTGF and VEGF, activate the activation of the YAP pathway, and upregulate the expression of Cx43 and Cyr61.

Schmidt et al. utilized the APPJ with Ar working gas to treat female mouse wound models for 20 seconds daily. Over 14 days, compared with the control group, the CAP group significantly accelerated the wound’s epithelial regeneration from day three to nine by quantitative analysis. They demonstrated that CAP could stimulate the migration of keratinocytes and fibroblasts *in vitro*, which resulted in the accelerated closure of gaps in scratch assays. The cause of this effect was related to the down-regulation of the gap junctional protein Cx43 mentioned above. Due to the down-regulation of E- cadherin, several integrins, and actin reorganization after CAP application, they confirmed that CAP could induce changes in adhesion junctions and cytoskeleton dynamics to accelerate wound healing (61). Lin utilized a DBD device with helium gas to treat the wound on the mice’s skin at 66 kHz and 6.0 kV. They found that galectin-1, as a β-galactose-binding lectin, was involved in many physiological functions to induce myofibroblast activation, which wound secret growth factors and chemoattractants to create new substrates and proteins in the extracellular matrix in wound healing. It was reported that galectin-1 accelerated wound healing by regulating the theneuropilin-1/Smad3/NOX4 pathway and ROS production in myofibroblasts (62). They examined the efficacy of low-temperature plasma on galectin expression in the skin using immunoelectron microscopy and found that the galectins increased quickly after the low-temperature plasma treatment and accelerated wound healing through the Smad pathway (6). Besides, CAP could increase the generation of fibroblasts and inner wall cells of blood vessels in wounds. The effect resulted from the link to the activity of some proteins, such as the vascular-associated migration cell protein (AAMP). CAP could strongly up-regulate the expression of the gene encoding AAMP, which could drive the RhoA–ROCK and Nod2–NF-κB signaling pathway to accelerate cell movement and promote wound healing (63). In summary, as **Figure 5** displayed, CAP promoted wound healing by enhancing the proliferation of keratinocytes, up-regulating the expression of cell growth-related genes, strengthening the release of cytokines, inhibiting wound microbial infection, and inducing the formation of blood vessels.

## ROS and RNS Attenuated ncISDs *Via* Keratinocytes’ Apoptosis, Immune Regulation, and Antioxidant Systems’ Accelerating

According to the lesions and pathological changes of skin diseases, ncISDs include nearly 100 different immune diseases caused by immune cells. For example, Th17 and ILC3 cells can lead to psoriasis characterized by neutrophil infiltration. Th2 cell can lead to eczema characterized by the abnormal infiltration of eosinophils. As for vitiligo, the CD8+T cell is critical, resulting in the high secretion of CXCL10, IFN-γ, and IL-17. These diseases have seriously affected the quality of patients’ lives and caused enormous socio-economic costs. Due to the genetic susceptibility of the body and the influence of the peripheral environments, ncISDs often lead to impaired epithelial function and pathological changes, including immune cell infiltration (64).

As a kind of complex hyperproliferative skin disease, psoriasis is an autoimmune disease. The typical clinical features are characterized by erythema, plaques, and scaly (65). In addition, excessive proliferation of keratinocytes, inflammation, infiltration, and pathological angiogenesis has been regarded as the typical pathological changes of psoriasis (66, 67). Our team used a DBD device with 0.06 W/cm^2^ to treat keratinocytes and evaluated the initial treatment effect of CAP on psoriasis by observing the death of keratinocytes and the release of related cytokines. We placed a petri dish containing HaCaT cells 10 mm below the surface DBD device. Then we explored the therapeutic effects of CAP on psoriasis by detecting the concentrations of ROS and RNS, observing the morphology, the viability, the apoptosis of keratinocytes, and the secretion of different cytokines, which played an essential role in the pathogenesis in psoriasis. From the results, we confirmed that CAP could suppress psoriasis. The main mechanisms of the results were exhibited: producing keratinocyte apoptosis, inhibiting excessive proliferation of keratinocyte and T lymphocytes, up-regulating the expression of IFN-γ and the mRNA of VEGF, increasing IL-6, IL-8, TNF-α, and VEGF release and decreasing the content of IL-12 (68). Lu et al. used cell and animal experiments to confirm CAP’s therapeutic function in psoriasis. They chose an APPJ device that flushed into helium and oxygen working gas with 15 kV and 1 kHz. Firstly, the cell model of psoriasis-like inflammation was simulated by adding LPS or TNF-α into HaCaT keratinocytes and then using a CAP-treated medium to cultivate HaCaT cells. Secondly, APPJ was directly applied to imiquimod-induced psoriasis-like dermatitis in mice. They observed that CAP increased ROS in HaCaT keratinocytes and caused keratinocyte apoptosis. Moreover, it was interesting that the IMQ-induced psoriasiform dermatitis gradually ameliorated lesions that displayed weakened epidermal proliferation and performed a thinner epidermal layer after APPJ treatment (69). Lee et al. indicated that CAP treatment might suppress psoriasis-like lesions in mice. Compared with the mice without CAP treatment, increasing epithelial thickness and the expression of pro-inflammatory molecules were inhibited in the skin of psoriasis-like mice treated with CAP. It has been well accepted that the expression of PD-L1 was relevant to the excessive activation of T cells. To their surprise, CAP could inhibit excessive activation of T cells *via* increasing PD-L1 expression in HaCaT cells (70). ROS and RNS from CAP could inhibit the excessive proliferation of keratinocytes and promote their apoptosis but restrain the proliferation of T lymphocytes and enhance the expression of PD-L1 protein, which was negatively correlated with the T lymphocytes’ proliferation. Moreover, ROS and RNS could also directly regulate the release of inflammatory factors such as IL-6, IL-12, and VEGF in lesions to achieve the effects of alleviating psoriasis.

**Figure 6:**
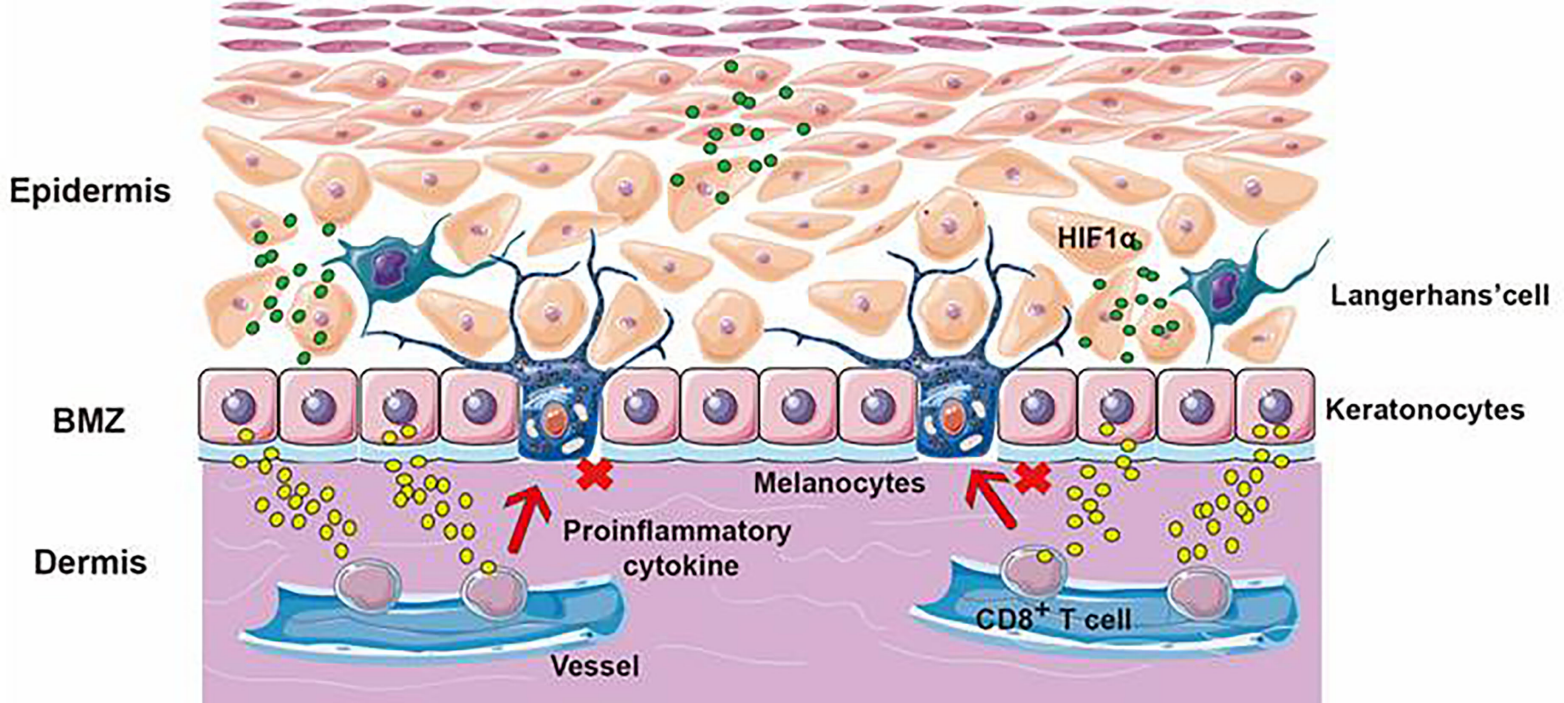
The application of CAP application on vitiligo. The skin is divided into the epidermis, dermis, and subcutaneous tissue layers. Vitiligo results from the targeted destruction of melanocytes. ROS and RNS from CAP could promote the release of HIF-1α, cause keratinocytes to release cytokines, inhibit the cytotoxicity of CD8^+^T cells and avoid the targeted destruction of melanocytes.

Vitiligo is the most frequent cause of depigmentation globally (71). Its standard treatment methods make vitiligo treatment challenging, owing to side effects, low cure, and high recurrence rates (72). Our team recently conducted experiments on CAP to treat vitiligo *in vitro *and *in vivo*. We used APPJ and DBD generating devices to treat vitiligo-like mice and patients with active focal vitiligo up to one month. With 8 kV and 9 kHz, CAP showed satisfactory efficiency in attenuating vitiligo. We used CAP-activated hydrogel and APPJ device to observe the interaction between CAP and vitiligo. From the vitiligo-like mouse, we documented that CAP could ameliorate vitiligo lesions and accelerate the distribution of follicular melanin. Furthermore, we confirmed that CAP could inhibit the infiltration of immune cells such as CD8^+^T cells, CD3^+^ cells, and CD11c^+^ cells, decrease the secretion of immune cytokines, and strengthen the responsibility of anti-oxidative systems. Moreover, we also observed the same changes in active focal vitiligo patients in the clinical trial without any side effects. In conclusion, we thought that CAP could inhibit the excessive activation of immune cells, restrain the release of inflammatory chemokines, induce the expression of Nrf2 and weaken the inducible nitric oxide synthase activity to attenuate vitiligo (73). From these studies, we considered that CAP ameliorated vitiligo by producing the generation of follicular melanocytes, inhibiting the excessive infiltration of immune cells, restraining the release of inflammatory cytokines, and regulating the activity of oxidative or anti-oxidative systems (**Figure 6**). As we all know, ncISDs are chronic and T-cell-associated inflammatory skin diseases. For psoriasis, with the characteristics of thickening and scaling, its pathogenesis was considered the proliferation of keratinocytes, infiltration of inflammatory or immune cells, and form of vessels. As for vitiligo, it involved oxidative stress pathways, immune inflammation, and the destruction of melanocytes. It could be known from the above documents that CAP attenuated ncISDs by promoting the apoptosis of keratinocytes, inhibiting the proliferation of keratinocytes, restraining excessive immune responses, regulating inflammatory cytokines, and reinforcing antioxidant systems.

## ROS and RNS Inhibited Cancer *Via* Producing Cell Apoptosis, Increasing Cytokines Release, and Activating the Different Pathways

Recent studies have suggested that researchers have concluded that the primary mechanism of CAP’s anti-cancer treatment might be related to the generation of ROS and RNS (74, 75). Free radicals, especially ROS and RNS, had been reported as the common mediator of apoptosis. ROS, RNS, and charged particles have been determined to be significant contributors to plasma-induced cell death (76). The excessive generation of ROS and RNS might damage cellular components, including DNA, proteins, and lipid membranes. However, most apoptosis pathways observed in CAP-treated cancer cells mainly depend on the injury of significant protein and DNA or the damage to the cell microenvironment.

Melanoma is considered as a highly invasive cancer that is highly resistant to most traditional chemotherapy and radiation therapies (77). Previous studies had provided several possible mechanisms by which CAP inactivated cancer cells, including ROS and RNS, charged particles, heat, pressure gradients, and changes in electrostatic and electromagnetic fields. In Keidar’s study, CAP had been translated into *in vivo* models of tumor treatment. From the results, they found that the mid-sized tumors in nude mice ameliorated after 2-min CAP treatment without any thermal damage (78). Chernets et al. utilized CAP application to treat melanoma in a mouse model, and the results confirmed that CAP could prohibit the activity and proliferation of melanoma. Their study showed that ROS, RNS, and its radicals from CAP created a chemistry response for eliminating subdermal B16 melanoma. They also indicated that the heat from CAP was insufficient to prohibit melanoma proliferation without the presence of reactive species (79). Ishaq et al. showed that CAP caused melanoma cells to over-expression TP73, which will lead to growth arrest and apoptosis (80). Ardnt et al. discovered that CAP treatment for 2 seconds could lead to irreversible cell cycle arrest, induction of phosphor-H2AX, and DNA damage (81, 82). In addition, Schneider et al. analyzed intracellular Ca^2+^ indicator fura-2 AM to analyze changes in cytoplasmic Ca^2+^ levels after CAP treatment of malignant melanoma cells. The results showed that CAP caused the acidification of water and solution, and the influx of Ca^2+^ in melanoma cells induced by CAP was enhanced in an acidic environment. Besides, NO induced by CAP was dose- and pH-dependent, and CAP-treated solutions could cause protein nitrification in cells under acidic conditions. Therefore, they believed that the decrease in pH caused by CAP treatment inhibited tumor cells’ proliferation and growth. The possible mechanism was that the synergistic effect of ROS, RNS, and acidic conditions produced by CAP affected the intracellular Ca^2+^ level of melanoma cells. Since the microenvironment of tumors was usually acidic, further acidification may be one reason for the specific anti-cancer effect of CAP (10). These results might indicate that CAP shared some similarities in their mechanism of action, possibly by causing ROS-dependent DNA or protein damage in melanoma. Of course, acidifying the microenvironment of tumor cells and enhancing the influx of Ca^2+^ to tumor cells could also cause the death of tumors.

Non-melanoma skin tumor primarily includes basal cell and squamous cell carcinoma. Basal cell carcinoma (BCC), most common in the head and neck region, is the most common form of skin tumor and 3 to 5 times more common than squamous cell carcinoma. Squamous cell carcinoma mainly occurs in the head and neck region and is more common in older men (83). Recently, Wang studied the effects and the related mechanisms of CAP-activated medium on cutaneous squamous carcinoma cells (SCC). They selected A431 epidermoid carcinoma cell lines and a CAP-activated PBS medium to incubate the A431 cells for 2 hours. Their results showed that the CAP-activated PBS medium could increase the intracellular ROS levels and inhibit the proliferation of A431 cells in a dose/time-dependent manner. At the same time, the CAP-activated PBS medium could lead to the apoptosis of A431 cells (84). Yang’s study aimed to explore the effects and related mechanisms of two CAP-activated solutions on TE354T cell and HaCaT keratinocyte cell lines. PAS was prepared by CAP irradiation of DMEM and PBS. Then they treated TE354T cells with PAS *in vitro* and evaluated the viability and apoptosis rate of TE354T cells and HaCaT cells. Western blotting and RNA sequencing were also detected. The results showed that CAP-activated PAS solution strengthened the apoptosis signaling in BCC, and the mechanisms were related to the activation of the different signaling pathways (85).

Besides the skin tumors mentioned above, CAP has shown remarkable effects on other kinds of tumors, such as the triple-negative breast cancers through hsa_circRNA_0040462 (86–88), glioblastoma multiforme (89), lung cancers (90), and pancreatic ductal adenocarcinoma (91). Recent studies confirmed that invivopen, a new source of CAP for cancer therapy, could ameliorate triple-negative breast cancer successfully. They found that CAP or PAM could inhibit the proliferation of triple-negative breast cancer, which showed a more significant inactivation on mesenchymal breast cancer than epithelial-derived breast cancer. Surprisingly, the above results also applied to the CAP inactivation of mesenchymal-derived bladder cancers (92, 93). In addition, tumor recurrence is often related to the rapid proliferation of remaining tumor cells in the body. In order to solve the threat of residual tumor cells, our team developed a new type of therapeutic plasma-activated biogel that could be implanted into mice to inhibit the proliferation of residual tumor cells. With 10 kHz and 7.5 kV, CAP could transform the solution into the plasma-activated biogel containing ROS with its temperature rising to 35°C. Then the plasma-activated biogel was placed in the tissue where the tumor cells were removed, and the tumor growth on the skin of the right-back of the mouse was monitored three times a week. The results showed that compared with other groups, the mouse in the plasma-activated biogel group was ameliorated entirely within two weeks. Moreover, the survival rate was more than six weeks, and no tumor recurrence had been seen. Through experiments, it could be known that the effect substances, such as H_2_O_2_, NO_2_^-^, NO_3_^-^, ONOO^-^ and O_2_^-^, could be released by plasma-activated biogel would inhibit the growth and recurrence of residual tumor tissues without any side effects (94). All in all, CAP could promote tumor cell apoptosis by activating the cell culture medium, acting on tumor cells, and directly inhibiting the proliferation of tumors. It could be seen from the above findings that the increase in the concentration of ROS and RNS in tumor cells was the key to the treatment of tumors by CAP. On the one hand, ROS and RNS could damage the DNA of tumor cells and the proteins such as tumor protein 73. On the other hand, the acidification of the tumor’s microenvironment and the increased concentration of Ca^2+^ in the microenvironment were also crucial in prohibiting tumor cells by CAP. In addition, the MAPK/JNK or NF-κB signaling pathway was also the main mechanism for CAP to inhibit the proliferation of tumors.

## Conclusion

**Figure 7:**
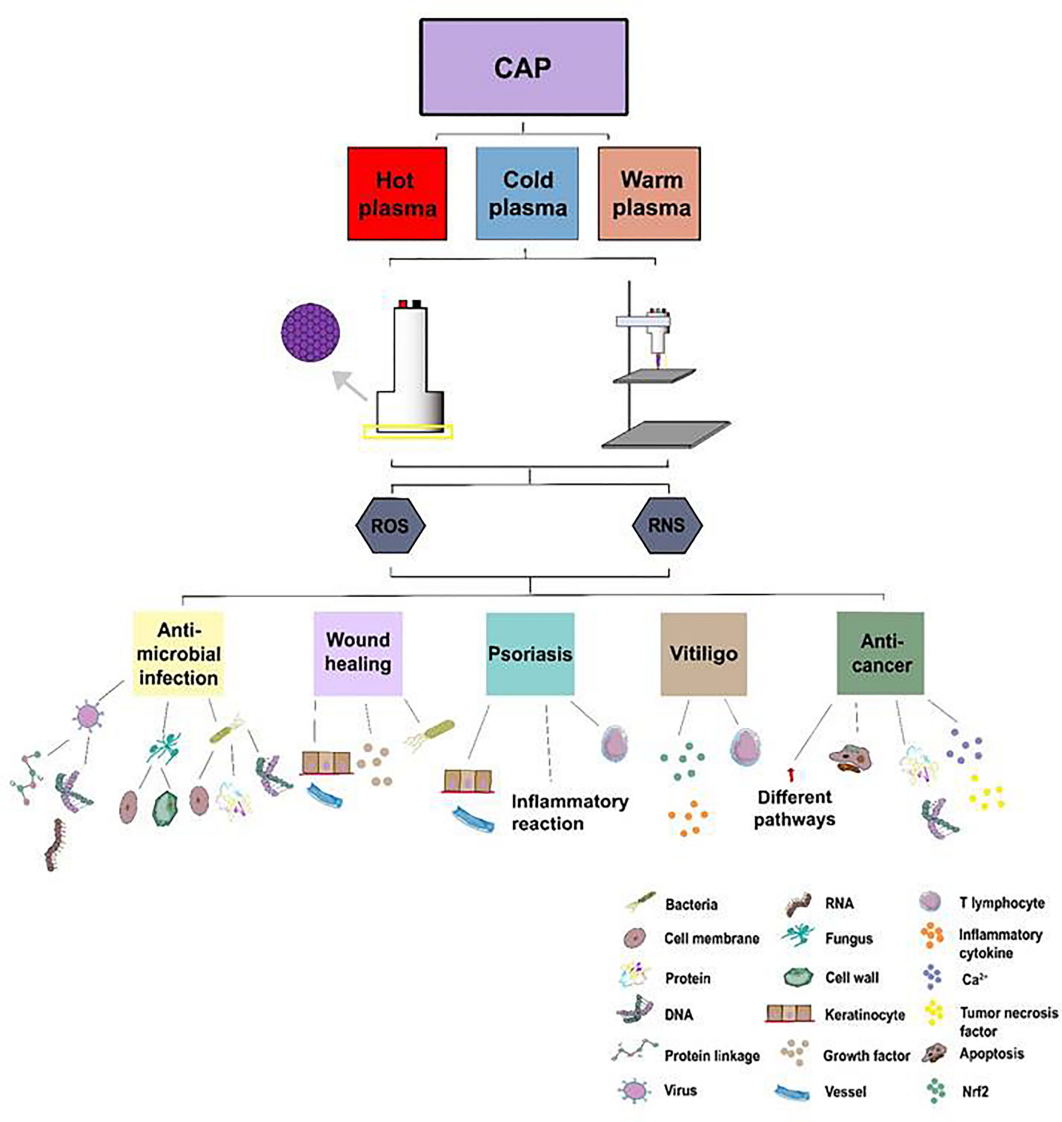
The treatment of CAP application on skin diseases. Atmospheric plasma has been divided into hot, warm, and cold atmospheric plasma. Two main kinds of CAP generators, DBD and APPJ, have been utilized to treat skin diseases such as inactivating microorganisms, accelerating wound healing, ameliorating ncISDs, and inhibiting tumors *via* ROS and RNS from CAP.

CAP has shown its apparent effects on different skin diseases such as infectious diseases, skin tumors, psoriasis, vitiligo, and wound healing. **Figure 7** has described the main mechanisms of CAP application on skin diseases. In terms of inhibiting microbial infections, with ROS and RNS, CAP destroyed biofilms or the cell membranes of microorganisms and damaged the DNA, RNA, or protein of microbes in a time or dose-dependent manner directly or indirectly; In wound healing, CAP could promote the proliferation of keratinocytes, enhance the expression of genes related to cell growth, and enhance the release of inflammatory factors. As for the ncISDs, CAP could promote the apoptosis of keratinocytes, inhibit the proliferation of keratinocytes, and suppress the excessive activation of T lymphocytes, which played a crucial role in psoriasis. CAP could also ameliorate the occurrence of vitiligo by reducing the excessive activation of the immune response and enhancing the ability of the antioxidant system. Finally, CAP could activate the apoptosis of cells, restrain the acidification of the cells’ microenvironment, increase intracellular Ca^2+^, and damage DNA to ameliorate melanoma. Last but not least, for the other tumor such as glioblastoma multiforme, lung cancers, and pancreatic ductal adenocarcinoma, ROS, and RNS could activate the apoptosis of the tumor and accelerate the release of the necrosis factors related to MAPK or NF-κB signaling pathways to inhibit tumors. As a new type of non-invasive physical tool, CAP has shown satisfactory results in medicine. It is believed that CAP will be applied to treat more skin diseases in the future and apply more therapeutic options for the treatment of skin diseases.

## Author Contributions

Y-mX and MK designed the manuscript. S-yZ wrote the manuscript. S-yZ and Y-mX created the figures. Y-mX and MK revised the manuscript. All authors contributed to the article and approved the submitted version.

## Conflict of Interest

The authors declare that the research was conducted in the absence of any commercial or financial relationships that could be construed as a potential conflict of interest.

## Publisher’s Note

All claims expressed in this article are solely those of the authors and do not necessarily represent those of their affiliated organizations, or those of the publisher, the editors and the reviewers. Any product that may be evaluated in this article, or claim that may be made by its manufacturer, is not guaranteed or endorsed by the publisher.
